# Sentinel surveillance of substance-related self-harm in Canadian emergency departments, 2011 − 19

**DOI:** 10.1186/s12889-022-13287-6

**Published:** 2022-05-14

**Authors:** Aimée Campeau, André S. Champagne, Steven R. McFaull

**Affiliations:** Public Health Agency of Canada, Ottawa, ON Canada

**Keywords:** Self-harm, Self-injury, Non-suicidal self-injury, Emergency department, Public health surveillance, Substance use, Self-poisoning

## Abstract

**Introduction:**

Self-harm is a public health concern that can result in serious injury or death. This study provides an overview of emergency department (ED) visits for patients presenting with substance-related self-harm.

**Methods:**

Cases of self-harm in the electronic Canadian Hospitals Injury Reporting and Prevention Program (eCHIRPP) database were extracted (April 2011 to September 2019; *N* = 15,682), using various search strategies to identify substance-related self-harm cases for patients 10 years and older. Cases involving alcohol, cannabis, illicit drugs, or medications (or any combinations of these) were included. Additional variables, including age and sex, location and the severity of injury (hospital admission) were examined. Proportionate injury ratios (PIR) were used to compare emergency department outcomes of self-harm and unintentional injuries involving substance use. Time trends were quantified using Joinpoint regression. For cases requiring hospital admission, text fields were analyzed for contextual factors.

**Results:**

A total of 9470 substance-related self-harm cases were reported (28.1% of all intentional injury cases), representing 820.0 records per 100,000 eCHIRPP records. While age patterns for both sexes were similar, the number of cases for females was significantly higher among 15-19 year olds. Over half (55%) of cases that identified substance type involved medications, followed by multi-type substance use (19.8%). In the ED, there were proportionally more treatments, observations, and admissions presenting with substance-related self-harm compared to substance-related unintentional injury cases. Among those aged 20+ years, a statistically significant increasing trend of 15.9% per year was observed, while among those aged 10-19 years a significant annual percent change of 16.9% was noted (2011 to 2019). Text field analysis demonstrated suicide attempt or ideation was a reoccurring theme among all age groups. Poor mental health status or conflict with family or an intimate partner were reported stressors, depending on age group. Additional self-harming injuries, such as cutting, were reported among all age groups.

**Conclusion:**

Our study found that hospital admission for substance-related self-harm was highest for patients aged 15-19 years, especially females, and that they were more likely to use medications. The statistically significant increasing trend of cases found between 2011 and 2019 is notable. Patients showed multiple types of adversities, demonstrating the complexity of this issue.

## Background

Self-harm, defined broadly as self-injurious behaviours both with and without suicidal intent that have non-fatal outcomes, [1] is a major public health concern. Self-harming behaviours including poisoning, cutting, burning, stabbing and others can result in repeated attempts of self-harm, serious injury or death [2, 3]. These behaviours are usually related to life stressors, or exposure to negative life events, and can cluster among some populations [4]. Significant portions of young people engage in self-harm worldwide [5]. Further, youth who self-harm are also more likely to experience adverse outcomes in early adulthood such as mental health problems and substance misuse [6].

Internationally, hospital-treated self-harm has increased in frequency, especially for young people [7]. Studies from the US, England, Ireland, and New Zealand have shown that for hospital-presenting or emergency departments (ED) cases of self-harm, self-poisoning is the most common mechanism reported by adolescents and young adults [7–11], while self-cutting has been reported as the most common method of self-harm in community samples [5]. In Canada, in fiscal year 2014/15 (April – March), hospitalizations associated with self-inflicted injuries were over three times higher than the number of suicides [12]. Among cases of self-inflicted injury, self-poisoning was the most frequent method used (86%), followed by cutting/piercing [12]. In Ontario rates of adolescent ED visits for both self-harm (including self-injuries and self-poisoning) and mental health concerns increased starting in 2009 and were higher and increased faster among females [13]. Another Ontario study found that about one in 100 ED presentations by youth were due to self-harm and that self-poisoning was also the method used most often followed by cutting [14]. While studies of hospital-presenting cases have been shown to underestimate the volume of presentations for self-harm [15, 16], identifying these trends, as well as the social, familial and individual factors that contribute to them, can support preventative policy and programming.

The objective of this study is to provide an overview of ED visits for patients presenting for substance-related self-harm in the electronic Canadian Hospitals Injury Reporting and Prevention Program (eCHIRPP) from 2011 to 2019. The term substance-related self-harm is used to indicate self-poisoning, including the intentional harmful use of both licit and illicit substances. The term non-suicidal self-injury (NSSI) is not used because NSSI excludes behaviours engaged with any level of suicidal intention [1], and eCHIRPP cases do not differentiate between suicidal and non-suicidal self-injury. This study aims to: (1) describe the demographic characteristics of presenting patients, place where the injury occurred, and severity of injury by sex; (2) compare emergency department outcomes of intentional self-harm and unintentional injuries involving substance use; (3) examine temporal trends (2011-2019) by age group and sex; (4) identify types of substances used by sex and age group and; (5) describe contextual factors reported in text fields for serious cases, by age group.

## Methods

### Data source

The eCHIRPP is a sentinel surveillance system that gathers injury and poisoning data from 19 selected EDs across Canada, 11 of which are primarily paediatric hospitals [17]. While the eCHIRPP database was not created to produce nationally representative incidence estimates of specific types of injuries and poisonings, it does serve as a data source capturing information on the circumstances surrounding such events, that is, how the injuries/poisonings occurred. Information regarding the circumstances under which the injury occurred is provided by the patient or accompanying caregiver, and clinical information is recorded by the attending physician or abstracted from the patient’s medical chart. Details regarding the development and uses of the eCHIRPP database have been published elsewhere [18].

### Case selection

**Fig. 1:**
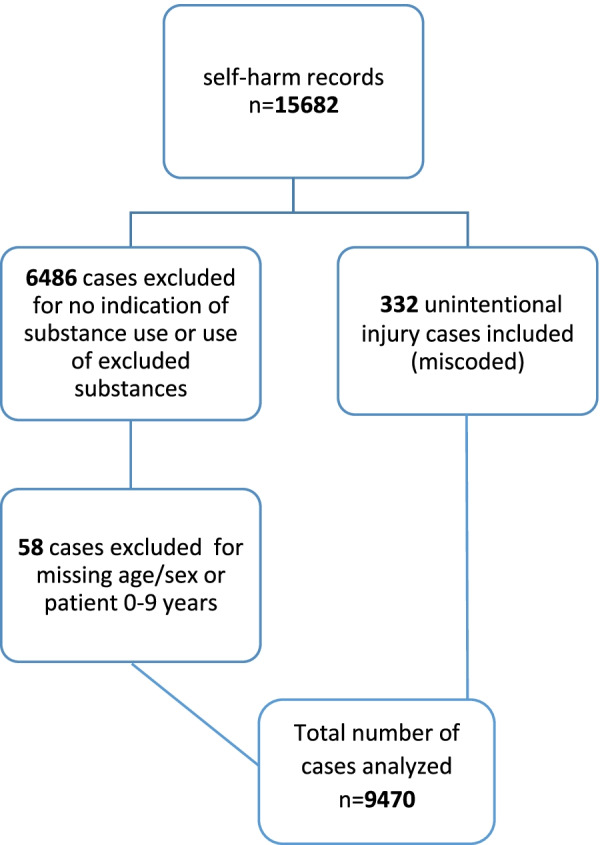
Case selection of substance-related self-harm in eCHIRPP, 2011-19

The eCHIRPP database includes cases of intentional injuries, including self-harm, assault and maltreatment, and unintentional injuries (i.e., accidental injuries). For our analysis, cases identified as intentional self-harm (hereafter “self-harm”) in the eCHIRPP database were extracted for all ages (*N* = 15,682) from April 2011 to September 2019. An additional search was conducted among unintentional injury cases (using keywords found in text fields of the confirmed intentional injury cases) to identify possible miscoded records and ensure good capture (*n* = 332). A variety of search strategies were used to identify substance-related self-harm using SAS 9.4 and Microsoft Excel 2016. The substance use and the substance ID variables were screened along with other variables and/or text fields to refine the sample (See Fig. 1). Cases involving alcohol, cannabis, illicit drugs, or prescription or over-the-counter medications, or any combinations of these, were included. Cases that reported the use of unknown substances (e.g. “pills”; “unknown drug”) were also included. Cases that reported the exclusive ingestion of poisons such as household cleaners (e.g. bleach, dish soap, laundry detergent), other chemicals (e.g. nail polish remover, hand sanitizer, windshield washer fluid, paint-thinner) or cases involving self-harm by carbon monoxide poisoning were excluded. Cases for which the date of birth and/or sex were unknown or the patient was under 10 years were also excluded (*n* = 58). It is standard practice that analysis of suicide and attempted suicide is restricted to those 10 years of age and older [19].

The following criteria were used to identify substances in self-harm cases: Alcohol use: (1) substance ID and/or (2) text fields containing terms like “alcohol”, without the use of other types of substances. Cannabis use: (1) substance ID and/or (2) text fields containing terms like “cannabis”, “marijuana” or “weed”, without the use of other types of substances. Illicit drug use: (1) substance ID and/or (2) text fields containing terms like “illicit drugs”, “cocaine”, or “heroin” without the use of other types of substances. Medication use (prescription or over-the-counter): (1) substance ID and/or (2) text fields containing terms like “medication”, “sleeping pills” or “acetaminophen”, without the use of other types of substances, although multiple medications may be present, including opioids. Multi-type substance use: (1) substance ID and/or (2) text fields containing different types of substances (e.g., alcohol and medications). Unknown substance: (1) substance ID and/or (2) text fields containing terms like “unknown drug” or “pills” without the use of other identified substances. A list of substances is available from the authors upon request.

### Statistical analysis

We conducted descriptive analyses to examine the distribution of characteristics of substance-related self-harm cases. Variables pertaining to person (sex and age), location where the injury occurred and the severity of the injury (observation or treatment in ED or admission to hospital) were obtained from the database. Hospital admission (including deaths) was used as a proxy for injury severity. We calculated means and interquartile range (IQR) for categorical variables for the full sample and for males and females. Furthermore, age and sex-adjusted proportionate injury ratios (PIRs) [20] concerning the disposition variable were calculated comparing the observed number of substance-related self-harm cases to those expected using the substance-related unintentional injuries as the referent population. Age specific proportions were calculated across seven age groups (10-14, 15-19, 20-29, 30-39, 40-49, 50-64 and 65+) and PIRs along with their respective 95% confidence intervals (CIs) for both males and females and sexes combined were calculated.

The data were normalized (dividing the number of cases by the total number in each age group, times 100,000) for each year and sex for two age groups: 10-19 years and 20 years and older. These are standard age groups for analysis due to the high number of pediatric cases in the eCHIRPP database. Joinpoint regression [21] was used to assess the trend by locating inflection points (joinpoints), if any, and calculating the Annual Percent Change (APC) of each identified segment according to the methods described by the National Cancer Institute [22]. Joinpoint regression software tests whether the APC of each segment is significantly different from zero at the α = 0.05 level and produces a 95% confidence interval (CI).

A random sample of 16% (*n* = 1547) of cases with a completed substance ID code were manually coded to identify categories of substance types outlined above (under Case Selection). Cases identified as “multi-type substance use” (e.g., alcohol and medications) were manually coded a second time to identify all substances. Therefore, the percentages in the multi-type substance category total more than 100 as the same case may be identified in more than one category.

### Text field analysis

For cases requiring hospital admission or resulting in death (i.e., serious cases), a content analysis methodology [23] was used to code text fields in order to better understand the contextual factors that contributed to self-harming behaviours. Cases were separated by age group (i.e., 10-14, 15-19, 20-49, 50+), and text fields were scanned to gain a broad sense of the type of information presented. Coding categories were developed after a more thorough reading of cases was conducted to identify recurrent behaviours, phrases and events. New categories were added or modified if a unique theme emerged after the initial categories were established (e.g., “bullying” only emerged as a risk factor in text fields for younger age groups). French text fields were re-checked by a fluent French speaker. Themes and descriptions of coded categories are presented along with frequencies.

## Results

### Case characteristics

A total of 9470 self-harm cases with substance use were reported to eCHIRPP between April 2011 and September 2019. These cases accounted for 28.1% of all intentional injury cases in eCHIRPP and 60.4% of all self-harm cases for this time-period.

Table 1 presents self-harm with substance use case characteristics by sex. The mean age was 22.8 years and the median age was 16.6. There were substantially more cases of self-harm with substance use for females (72.4%) than for males (27.6%). Age patterns for females and males were similar, however, the number of cases for females tended to be higher especially for those aged between 15 and 19 years (*n* = 3543 vs *n* = 906). The second highest frequency of cases for females was among the 10-14 year age group, while for males it was among the 20-29 year age group. Males aged 30-39 years and 50-64 years had a slightly higher frequency of self-harm cases with substance use than females. Of all self-harm cases with substance use, 36.5% resulted in admission to hospital with a higher proportion among females (39.2%) than males (29.5%). Eight patient fatalities were reported either on arrival to hospital or after the patient was admitted. Of the cases where a location was specified, 87.4% reported that the injury took place in a private home.

**Table 1:** Characteristics of self-harm with substance use by sex, eCHIRPP, 2011 to 2019

Characteristic	Total	Males n (%)	Females n (%)
Total	9470	2611 (27.6)	6858 (72.4)
Age (years)			
Mean	22.8	29	20.4
Median (IQR)	16.6 (15.1-23.9)	21.6 (16.1 - 39.8)	16.2 (14.8 - 17.8)
Age group (years)			
**10-19**	**6651 (70.2)**	**1227 (47.0)**	**5424 (79.1)**
10-14	2202 (23.3)	321 (12.3)	1881 (27.4)
15-19	4449 (47.0)	906 (34.7)	3543 (51.7)
**20+**	**2819 (29.8)**	**1384 (53.0)**	**1434 (20.9)**
20-29	926 (9.8)	394 (15.1)	532 (7.8)
30-39^a^	645 (6.8)	342 (13.1)	302 (4.4)
40-49	503 (5.3)	246 (9.4)	257 (3.8)
50-64	609 (6.4)	338 (13.0)	271 (4.0)
65+	136 (1.4)	64 (2.5)	72 (1.1)
Disposition^b^			
Left without being seen or advise only	503 (5.3)	150 (5.8)	353 (5.2)
Treated in ED, follow up as needed	1216 (12.9)	375 (14.4)	841 (12.3)
Treated in ED, follow-up required	1367 (14.4)	346 (13.3)	1021 (14.9)
Observed in ED, follow-up as needed	1853 (19.6)	719 (27.6)	1134 (16.6)
Observed in ED, follow-up required	1057 (11.2)	246 (9.4)	811 (11.8)
Admitted to hospital	3454 (36.5)	769 (29.5)	2685 (39.2)
Dead on arrival or after being admitted	8 (0.08)	-	-
Location^c^			
Private home	5028 (53.1)	–	–
Institutional home	184 (1.9)	–	–
School or university	192 (2.0)	–	–
Highway, road, alley, bridge etc.	119 (1.3)	–	–
Public park	55 (0.6)	–	–
Hospital, community health center, other health services	33 (0.3)	–	–
Shop or shopping centre, commercial eating space	53 (0.6)	–	–
Hotel	26 (0.3)	–	–
Other specified location	61 (0.6)	–	–
Unspecified	2798 (29.5)	–	–

^a^1 case had no information on sex for this age category

^b^12 missing cases for disposition

^c^921 cases missing for location

### PIR analysis

Table 2 presents age and sex-adjusted proportionate injury ratios (PIR) comparing substance-related self-harm with substance-related unintentional injuries and poisonings by the disposition variable. As shown, overall there were proportionally more treatments (PIR = 18.0, 95% CI: 17.1–18.9), observations (PIR = 29.3, 95% CI: 27.6–31.0, and admissions (PIR = 48.0, 95% CI: 46.4–49.5) requiring medical follow-ups among patients presenting with substance-related self-harm compared to the referent population.

**Table 2:** Adjusted proportionate injury ratios (PIR): substance-related self-harm and substance-related unintentional injuries, by disposition, 2011-19

Characteristic	All		Males		Females	
	PIR	95% CI	PIR	95% CI	PIR	95% CI
Disposition						
Treated in ED, follow up as needed	7.3	(6.9 -7.7)	6.3	(5.8 - 7.0)	6.8	(6.4 - 7.3)
Treated in ED, follow-up required, referred	18.0	(17.1 - 18.9)	11.4	(10.3 - 12.6)	22.2	(20.9 - 23.5)
Observed in ED, follow-up as needed	16.4	(15.8 - 17.1)	23.4	(21.9 - 24.9)	11.3	(10.7 - 12.0)
Observed in ED, follow-up required	29.3	(27.6 - 31.0)	33.0	(29.2 - 37.3)	22.6	(21.1 - 24.1)
Admitted to hospital	48.0	(46.4 - 49.5)	22.1	(20.6 - 23.7)	68.3	(65.9 - 70.8)

A similar finding was noted when examining each sex. There were proportionally more treatments, observations and admissions in relation to the referent population, though the magnitude of PIRs varied by sex and level of care (see Table 2). Particularly large PIRs were observed regarding admissions for both males and females, though the magnitude was more pronounced among the latter: whereas a PIR of 68.3 (95% CI: 65.9–70.8) was noted among females, a PIR of 22.1 (95% CI: 20.6–23.7) was observed among males. More modest though significant PIR values were observed when examining treatments in EDs requiring follow-ups as needed among both sexes –males (PIR = 6.3, 95% CI: 5.8–7.0) and females (PIR = 6.8, 95% CI: 6.4–7.3).

### Time trend analysis

**Fig. 2:**
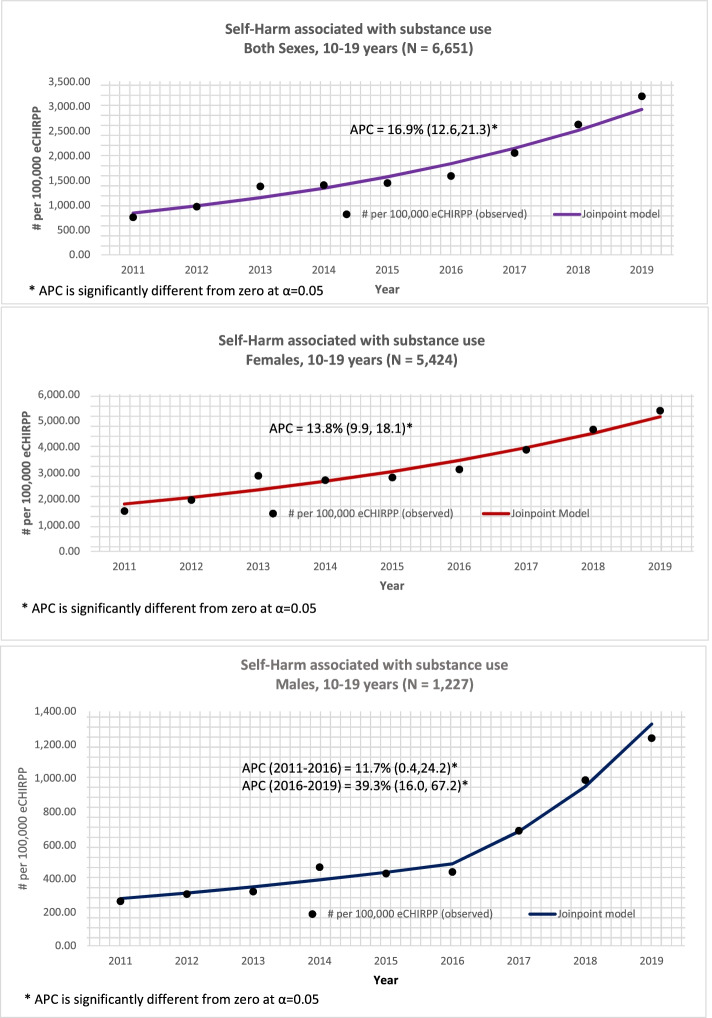
Joinpoint regression analysis for substance-related self-harm for 10-19 year olds by sex, 2011 to 2019

**Fig. 3:**
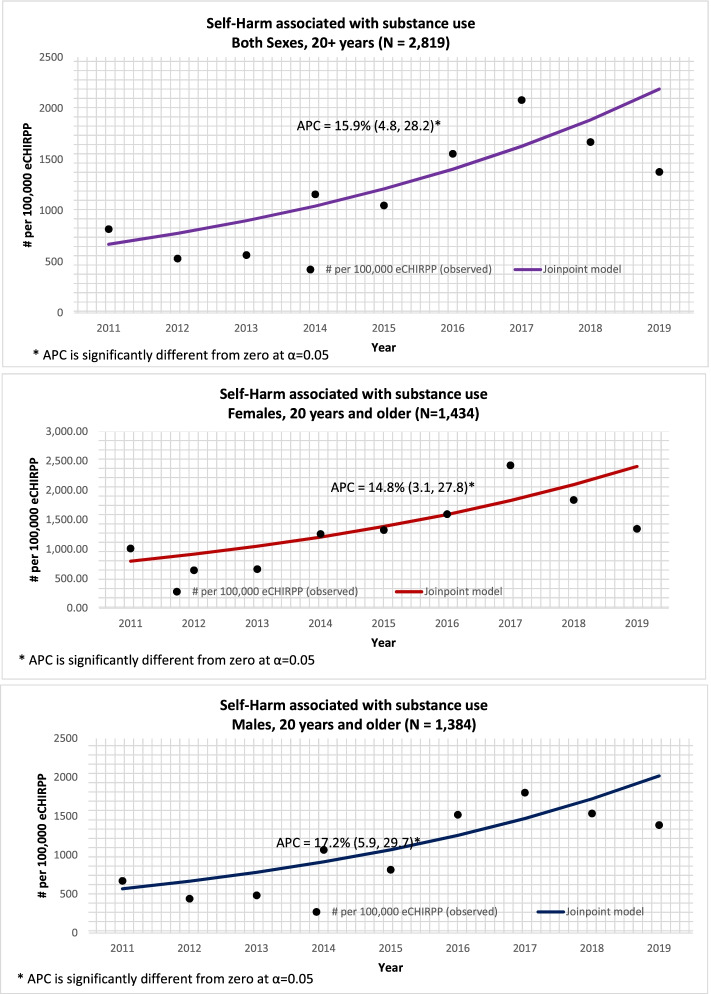
Joinpoint regression analysis for substance-related self-harm for 20+ year olds by sex, 2011 to 2019

Figure 2 shows the results of the Joinpoint regression analysis of substance-related self-harm cases for both sexes among 10-19 year olds. Between 2011 and 2019, there was a significantly increasing trend among youth 10-19 years of age – at 16.9% per year. Among males there was a significant increase (APC = 11.7%) between 2011 and 2016 and a sharp significant increase from 2016 to 2019 (APC = 39.3%). Among females 10-19 years, there was a significant increase of 13.8% between 2011 and 2019. Figure 3 shows the results of the Joinpoint regression analysis of substance-related self-harm cases for both sexes among those 20 years and older. Among those aged 20 years and older, the trend was similar for both sexes. Overall, between 2011 and 2019, there was an increasing and significant trend of 15.9% per year.

### Substance type

Tables 3 and 4 present types of substances used by sex and age group. Over half (55%) of cases that identified the type of substance used involved only medications (prescription and/or over-the-counter), followed by multi-type substance use (19.8%). Of cases with multi-type substance use, both alcohol and cannabis were more likely to be used with other substances than on their own by both males and females. Of cases with multi-substance use, males were more likely than females to use illicit substances with other types of substances (38.1% vs 15.0%, *p* < 0.005) and, although not significant, females tended to use medications with other types of substances more than males (75.7% vs 57.5%). Youth 15-19 years, followed by youth 10-14 years, were most likely to use medications to self-harm. Multi-type substance use was also most frequently used by youth 15-19 years and remained the most frequently used substance for adults 20-49 years. The exclusive use of alcohol was reported more often than the exclusive use of cannabis or illicit drugs among all age groups.

**Table 3:** Substance-related self-harm by substance type and sex

Substance	Totals n (%)	Males n (%)	Females n (%)
Total	1547 (100)	458 (29.6)	1089 (70.4)
Alcohol only	170 (10.9)	87 (19.0)	83 (7.6)
Cannabis only	26 (1.7)	9 (2.0)	17 (1.6)
Illicit only	81 (5.2)	41 (9.0)	40 (3.7)
Medications^a^ only	851 (55.0)	159 (34.7)	692 (63.5)
Unknown	112 (7.2)	28 (6.1)	84 (7.7)
Multi-type substance	307 (19.8)	134 (29.3)	173 (15.9)
Included alcohol^b^	257 (83.7)	116 (86.6)	141 (81.5)
Included cannabis	64 (20.8)	32 (23.9)	32 (18.5)
Included illicit	77 (25.1)	51 (38.1)	26 (15.0)
Included medications	208 (67.8)	77 (57.5)	131 (75.7)

^a^Prescription and non-prescription

^b^Percentages under the multi-type category total more than 100 has the same case is identified in more than one category

**Table 4:** Substance-related self-harm by substance type and age group

Age Group (years)	Total	Substance Type					
		Alcohol	Cannabis	Illicit drugs	Medications	Unknown	Multi-category
Total	1547 (100)	170 (11)	26 (1.7)	81 (5.2)	851 (55)	112 (7.2)	307 (19.8)
**10-19**	**1018 (65.8)**	**61 (35.9)**	**24 (92.3)**	**21 (25.9)**	**695 (81.7)**	**102 (91.1)**	**115 (37.5)**
10-14	321 (20.7)	20 (11.8)	–	–	246 (29.9)	30 (26.8)	19 (6.2)
15-19	697 (45.1)	41 (24.1)	21 (80.8)	18 (22.2)	449 (52.8)	72 (64.3)	96 (31.3)
**20+**	**529 (34.2)**	**109 (64.1)**	**–**	**60 (74.1)**	**156 (18.3)**	**10 (8.9)**	**192 (62.5)**
20-29	179 (11.6)	31 (18.2)	–	20 (24.7)	55 (6.5)	6 (5.4)	66 (21.5)
30-39	116 (7.5)	25 (14.7)	–	22 (27.2)	23 (2.7)	–	44 (14.3)
40-49	95 (6.1)	20 (11.8)	0	7 (8.6)	30 (3.5)	0	38 (12.4)
50-64	117 (7.6)	24 (14.1)	0	11 (13.6)	41 (4.8)	–	39 (12.7)
65+	22 (1.4)	9 (5.3)	0	–	7 (0.8)	–	5 (1.6)

- suppressed due to small cell count

### Text field analysis

Table 5 provides an analysis of text fields of cases admitted to hospital, by age group. Suicide attempt or ideation was a major reoccurring theme among all age groups and was reported in 52.2% of all cases. Poor mental health status (e.g., feeling “upset”, “depressed” or “stressed”) was also frequently reported, especially among youth aged 10-19 years. Conflict with family member(s) was a common stressor among youth aged 10-19 years, and conflict with or separation from an intimate partner was reported more often among older youth aged 15-19 years and older age groups. Other stressors specific to youth aged 10-19 years included conflict with friends or peers, bullying and assault or abuse. Pre-existing conditions (e.g., PTSD or diagnosed depression or anxiety disorder), and history of self-harming behaviours, were also notable themes among youth. A small number of patients reported an inability to cope with chronic pain or illness and only emerged among adults aged 50+ years. The additional use of objects for self-inflicted injuries, such as cutting/lacerations, were reported in text fields among all age groups (ranging from 4.5-6%). Stabbing, although not common, was notable among adults 20-49 years. Hanging was also not common (1%), but cases with substance use and hanging accounted for the majority of fatalities.

**Table 5:** Contextual factors for patients admitted to hospital for substance-related self-harm (by age group)

**Theme**	**Description**	**Frequency n (%)**				
		Youth 10-14 years *n* = 888	Youth 15-19 years *n* = 1795	Adults 20-49 years *n* = 536	Older Adults 50+ *n* = 243	Total *n* = 3462
Suicide attempt	Suicidal attempt, gesture, ideation, or stated “wanted to die”	489 (55.1)	937 (52.2)	234 (43.7)	148 (60.9)	1808 (52.2)
Stressors	Feeling: “upset”; “depressed”; “stressed”; overwhelmed”; “sad”	183 (20.6)	354 (19.7)	52 (9.7)	16 (6.6)	605 (17.5)
	Conflict/separation with partner	10 (1.1)	74 (4.1)	49 (9.1)	7 (2.9)	140 (4.0)
	Conflict with a family member	67 (7.5)	82 (4.6)	14 (2.6)	9 (3.7)	172 (5.0)
	Conflict with friend/peer	9 (1.0)	18 (1.0)	-^a^	–	29 (0.8)
	Assault/abuse	9 (1.0)	11 (0.6)	–	–	24 (0.7)
	Bullying	12 (1.4)	9 (0.5)	0	0	21 (0.6)
	Death of loved one	–	6 (0.3)	–	–	12 (0.3)
	Inability to cope with illness or pain	0	–	–	6 (2.5)	7 (0.2)
Pre-existing mental health condition	“history of depression” or other diagnosis	21 (2.4)	95 (5.3)	10 (1.9)	0	126 (3.6)
	Past attempt/history of self-harming behaviours	28 (3.2)	45 (2.5)	0	0	73 (2.1)
	Eating disorder	–	–	0	0	5 (0.1)
Self-harm with an object^b^	Cutting/lacerations	40 (4.5)	105 (5.8)	32 (6.0)	13 (5.3)	190 (5.5)
	Stabbing	–	–	16 (3.0)	–	24 (0.7)
	Hanging	9 (1.0)	11 (0.6)	–	–	29 (0.8)
	Shooting	0	0	–	–	7 (0.2)
	Two or more	–	–	–	–	10 (0.3)

^a^suppressed due to small cell count

^b^some patients also burned themselves but these cases were supressed due to small cell count

## Discussion

The aim of this study is to describe cases of self-harm with substance use from April 2011 to September 2019, based on eCHIRPP data. The mean age of patients in this study was relatively young but consistent with other eCHIRPP studies, given that the majority of participating hospitals are paediatric [24]. As previously reported, both nationally and internationally, our study found that adolescent females accounted for the majority of cases presenting to the ED with self-harm with substance use [5, 8, 10, 12, 25]. Similar to findings presented by Canner et al., (2018) [10] our study found that while the overall age group distribution for males and females were similar, the percentage for females was much higher. Possible reasons for this are that women attempt suicide more frequently than men, peaking in mid-adolescents, while men are more likely to die by suicide due to the use of more violent methods [10, 26]. Non-substance-related suicides may be more likely to bypass the ED and not be captured in eCHIRPP. Another potential reason is that females who misuse substances are more likely to be injured, and present to hospital, as compared to males [27]. Fatalities among our sample were low; however, it is worth noting that the majority of cases, for both sexes, required admission to hospital indicating the severity of the self-harming behaviour. Overdose, whether intentional or unintentional, can lead to irreversible organ damage such as kidney and liver failure [28, 29] with poorer outcomes typically reported in older patients [30].

As demonstrated in previous studies [31], medications (both prescription and non-prescription) were used most frequently by both sexes. A study examining prescription medication availability found that patients who engaged in deliberate self-poisoning had a greater prescribed medication load than the general population [32]. Cases involving prescription medications sometimes included opioids; however, the eCHIRPP database is not able to report whether the ingested opioid was prescribed to the patient or prescribed to someone else in the household (e.g., belonging to a parent). Given that opioid-related deaths and hospitalizations have been increasing in Canada [33], the safe storage and disposal of medications, even in households with older children, may be a necessary prevention strategy [8]. Other strategies include restricting packet size of over-the-counter medications, restricting the sale of medications to pharmacies (i.e., not other retail outlets), and restricting sales to persons 18 years and over [34, 35]. Following medications, the use of multiple types of different substances were used most frequently, with alcohol dominating as a co-occurring substance in this category. History of alcohol use with other mechanisms of self-harm has been noted in other studies [34]. Further, alcohol use disorder has been associated with an increased risk of suicide ideation, suicide attempt and death by suicide [36]. Men were more likely than women to use illicit drugs in combination with other types of substances. Overall, cannabis was more likely to be used with other substances than in isolation. A previous study found that, medications and illicit drug use were significantly associated with hospitalizations while cannabis was not [37].

The time trend analysis demonstrates a significant trend among both sexes and all ages, particularly since about 2016. Part of this increase may be surveillance artifact due to improved capture and/or increased reporting (see limitations section). A relatively recent increase in awareness of mental health issues, and a reduction of the stigma associated with mental health, through national initiatives and social media campaigns around 2011 [38] may also account for part of this increase. It is also worth noting that the time-period of this study is pre-COVID-19 pandemic period. There is some evidence that substance use and self-harm have increased during the pandemic [39], and the results of this study represent a baseline for comparison in future studies (which are currently underway).

The proportion of observations, treatments and admissions were significantly higher among patients presenting to the ED with substance-related self-harm injuries and poisonings compared to the referent population, as noted in our PIR analysis. Two important considerations may help explain our results. First, the magnitude of these findings are reflective of the large proportional difference of the presence of substances among self-harm cases compared to those categorized as unintentional. Specifically, while the majority (58.5%) of all self-harm cases documented the presence of one or more substances – a finding consistent with previous studies [10, 40, 41] – the presence of one or more substances among all cases categorized as unintentional injuries and poisonings was reported in only 2.8% of cases (data not shown). Such a large discrepancy can contribute in magnifying the PIRs, even while adjusting for age and sex.

The second consideration takes into account the nature of these cases, whereby the deliberate self-harm by patients may in some instances warrant a greater need for medical attention compared to those who unintentionally or recreationally consumed substances. For instance, in our study, prescribed and/or over-the-counter accounted for the majority (55%) of all substance-related self-harm cases. This finding may be indicative of the clinical severity of such cases, as evidenced by a number of studies highlighting the acute health complications of prescription and over-the-counter medication overdoses [42–44] and thereby offering an explanation of the noted large PIRs in all levels of care, especially those regarding hospital admission, a level reflecting the need for further treatment and/or observation.

Additionally, though the quantity and dosage of ingested substances are not regularly reported in eCHIRPP records, a recent national study conducted in Ireland may also help provide insight on the gravity of consumption patterns of self-harming patients presenting in health care settings. Results of Cully et al.’s study [45] showed that, when specified, the majority of self-harming patients presenting to the ED consumed in the range of 20 or more tablets of prescription or over the counter medications or illicit substances. Though dose and substance dependent, such excess may have serious health implications requiring important medical intervention. If similar consumption patterns are found among the Canadian population, it may offer insight of the noted PIRs observed in our study, all pointing to higher proportions of observations, treatments and admissions among patients presenting to the ED with substance-related self-harm injuries and poisonings compared to the referent population. A Canadian study comparing detailed consumption patterns among substance-related self-harm cases and substance-related unintentional harm cases may help shed light in this area.

Text fields revealed additional notable findings. A high percentage of cases in each age group reported that the self-harming event was either an attempted suicide or included suicidal ideation. As reported in other studies, ED text fields involving substance use often included descriptions of depression, or feelings of sadness or being overwhelmed [24, 37]. Exposure to difficult life-events can lead to self-harm [4]. Stressful interpersonal events, such as conflicts with family members, conflict or a breakup with a partner, assault or bullying were reported as influencing factors leading up to the self-harming event. The capacity of people to cope with stressors can vary by age, sex and their ability to access supports [26]. Further, due to their developmental stage, adolescents may be more susceptible to reacting to negative social interactions and cues than adults [4]. Consistent with other studies [10, 25, 46], some cases identified the presence of a pre-existing mental health condition such as depressive or anxiety disorders, especially among youth. Although not a significant number of cases reported past or repeated self-harm in our study, it has been identified as a risk factor for eventual suicide [2, 47]. A systematic review found that the incidence of fatal repeat self-harm was 1.6% of patients within 1 year, increasing to 3.9% by 5 years and 4.2% at 10 years [3].

While our study did not capture a large sample of older adults, other studies have reported a high incidence rate of suicide related ED visits made by older adults involving the use of drugs, alcohol or both [48]. Later-life mental health and substance use disorders are risk factors for self-harm in older adults presenting to the ED, and contribute to outcomes that require more intensive and long-term health care services for this population [49]. Although less information exists on self-harm and middle aged adults, data from the US indicates that suicides among 35-64 year olds increased between 1999 and 2010 and self-poisonings were the second most common method used [50].

Finally, previous ED studies have demonstrated that cutting carries a greater risk of repetition and eventual suicide than poisoning, among adolescents [4]. Of those admitted to hospital, our study found cutting (and less often stabbing) accompanied substance use as an additional self-harming mechanism in cases among all age groups, and was reported in cases with reported suicide attempt and suicidal ideation. This may be considered a notable finding given that debate exists as to whether self-harming behaviours such as cutting should be exclusively categorized as a non-suicidal self-inflicted injury [51].

### Strengths and limitations

The eCHIRPP database provides on-going, timely and detailed clinical data from 19 ED across Canada and is a good source for examining trends in the pediatric population. Information from eCHIRPP text fields provides unique information regarding circumstances surrounding the injury event that highlight potential risk factors for injuries. For the qualitative component of this study, all cases where the patient was admitted to hospital were coded individually, including text fields.

There are a number of limitations of our study. As eCHIRPP is a sentinel surveillance system, it is not representative of the Canadian population. However, Pickett et al. found that CHIRPP may be representative of general youth injury patterns in Canada [52]. Older teenagers and adults who are seen at general hospitals, First Nations, Inuit, Métis and those who live in rural and remote areas are underrepresented in the eCHIRPP database [17]. The eCHIRPP is a dynamic surveillance system with cases entered daily at each of the 19 sentinel hospitals.

Some of the increases seen in the Joinpoint analysis for this study may be surveillance artifact. Some ED sites have improved their capture of self-harm cases faster than others. Also, recent increased awareness and promotion of help-seeking for mental health issues may have resulted in increased presentation to the ED. In both situations, it is prevalent cases that are being revealed and entering the ED system, rather than an increase in incident cases.

eCHIRPP is generally a poor source of fatality data. Because cases identified as self-harm in eCHIRPP included injuries due to both suicidal and non-suicidal intent, fatalities are a possible outcome. The eight fatalities found in our study could be underestimates. Further, some of the fatalities were identified through text fields (and not the disposition category) which may be problematic given that this practice is not consistent among all eCHIRPP sites.

As mentioned above, the eCHIRPP does not differentiate between suicidal and non-suicidal self-injury. Therefore, the number of cases of self-harm identified as the result of suicide attempt could be underrepresented. As our analysis was restricted to substance-related self-harm, our results may lack generalizability for self-harm cases. The information provided in text fields vary based the patient’s or caregiver’s ability to convey their account of events which can be impacted by the severity of their injuries, among other factors. Therefore, eCHIRPP does not fully capture variables identified in the text field analysis of those presenting in ED, such as past medical history. Finally, our sample is limited to those who came to the ED and does not account for those who self-harm and do not seek treatment.

## Conclusions

Self-harm is a public health concern that requires our attention. Substance use, along with mental health issues, are proven risk factors for long-term negative health outcomes and for suicide. Our study found that hospital admission was highest for youth aged 15-19 years, especially females, and that they were more likely to use medications (prescription and over-the-counter). Although substance use was highlighted in this study patients presented with multiple types of adversities, at varying levels of severity, demonstrating the complexity of this issue. By continuing to monitor trends, and report risk factors as experiences by those who self-harm, we can help inform effective prevention efforts and treatment.

## Data Availability

Data for CHIRPP are publicly available http://www.phac-aspc.gc.ca/injury-bles/chirpp/index-eng.php by contacting the Centre for Surveillance and Applied Research, (Injury.Surveillance@phac-aspc.gc.ca).

## Abbreviations

CHIRPP: Canadian Hospitals Injury Reporting Prevention Program
PHAC: Public Health Agency of Canada
ED: Emergency department
APC/AAPC: Annual Percent Change**/**Average Annual Percent Change
